# Implementing nursing interventions based on stress system theory alongside painting therapy for pediatric post-traumatic stress disorder following accidental injury

**DOI:** 10.1186/s12912-024-02159-6

**Published:** 2024-07-18

**Authors:** Xing Yuan, Bing Xu, Bao Cai, Shan Huang, Kai-Li Jiang

**Affiliations:** Department of Pediatric Surgery, Anqing Municipal Hospital, NO. 87 Tianzhushan East Road, Anqing, Anhui Province 246000 China

**Keywords:** Accidental injury, Coping style, Painting therapy, Post-traumatic growth, Post-traumatic stress disorder, Quality of life, Stress system theory

## Abstract

**Objective:**

The aim of this study is to examine the impact of a nursing intervention based on stress system theory, coupled with painting therapy, on children experiencing post-traumatic stress disorder (PTSD) subsequent to an accidental injury.

**Methods:**

The clinical data of 100 children diagnosed with PTSD following accidental injuries were retrospectively analyzed for the period spanning April 2021 to May 2023. There were 48 children who received standard nursing care between April 2021 and April 2022 in the control group, and 52 children who received nursing intervention based on stress system theory combined with painting therapy between May 2022 and May 2023 in the observation group. Scores of PTSD Self-evaluation Scale (PTSD-SS), post-traumatic growth, coping style, quality of life, and family satisfaction were compared between the two groups.

**Results:**

Prior to nursing care, the scores of each dimension in the PTSD-SS, post-traumatic growth, coping style, and quality of life were similar between the two groups (*P* > 0.05). Following nursing intervention, the observation group exhibited lower scores in each dimension of the PTSD-SS compared to the control group. Moreover, the scores in each dimension of the children’s version of the Post-Traumatic Growth Inventory (PTGI) were higher in the observation group than in the control group. Additionally, the Confrontation scores in the Medical Coping Modes Questionnaire (MCMQ) were higher in the observation group than in the control group, while the scores of Avoidance and Resignation were lower in the observation group than in the control group. The scores of each dimension in the Pediatric Quality of Life Inventory Measurement Models (PedsQL4.0) were higher than those in the control group (*P* < 0.05), and the family satisfaction in the observation group (96.15%) was higher than that in the control group (81.25%), with *P* < 0.05.

**Conclusion:**

The implementation of nursing intervention based on stress system theory combined with painting therapy in children with PTSD following an accidental injury can alleviate stress, help them actively cope with the condition, promote post-traumatic growth, and improve the quality of life and family satisfaction.

## Introduction

Due to their young age, high activity levels, and limited self-protection awareness and ability, children are susceptible to experiencing accidental injuries in both their day-to-day lives and recreational activities. These injuries may include fracture, traffic accidents, and falling [1]. Accidental injuries not only cause physical harm to children, but also increase their psychological stress and trigger post-traumatic stress disorder (PTSD), which can persist for days or years, and severely affect the quality of life in children [2]. Post-traumatic growth refers to the positive growth changes of an individual after experiencing trauma. With the improvement in the living standards of individuals and the development of positive psychology, patients have higher expectations for clinical care. As a result post-traumatic growth has become a topic of research in recent years. [3] According to the stress system model theory, the factors related to psychological stress involve stress stimuli, stress response, and coping style. The process of stress response is ultimately determined through the mediating role of these factors. It is of great significance to take specific nursing interventions accordingly to alleviate stress disorder. Alternative methods such as art therapy [4, 5] and animal assisted intervention [6, 7] have been widely used in reducing the stress response. Painting therapy for children is a therapeutic approach that utilizes visual art forms to help children express themselves and explore their emotions in a supportive and non-verbal manner. Through the creative process, children can communicate thoughts and feelings that might be challenging to express verbally. Painting therapy provides a medium for self-expression, self-discovery, and emotional processing, fostering overall well-being. This approach has been widely applied in many fields [8]. Building upon this premise, we performed a retrospective analysis of clinical data from 100 children diagnosed with PTSD subsequent to accidental injuries, aiming to assessing the application efficacy of a nursing intervention based on stress system theory, complemented by painting therapy. We hypothesize that children receiving the combined intervention will show significant improvements in PTSD symptoms, post-traumatic growth, coping styles, quality of life, and family satisfaction compared to those receiving standard care.

## Data and study method

### General data

A retrospective analysis was conducted on the clinical data of 100 children with PTSD following accidental injury. These children were admitted to the Anqing Municipal Hospital in Anhui Hospital between April 2021 and May 2023. During hospitalization, children and their legal guardians were given the option to choose between receiving routine nursing care alone or routine nursing care combined with stress system theory-based intervention alongside painting therapy. The study involved 48 children who received only routine nursing care between April 2021 and April 2022, constituting the control group. In contrast, 52 children who received nursing intervention incorporating stress system theory alongside painting therapy between May 2022 and May 2023 formed the observation group. There were 26 male children and 22 female children, aged between 6 and15 years, with an average age of (10.27 ± 1.22) years in the control group. The types of accidental injuries experienced in this group were: 12 cases of fracture, 20 cases of car accidents, and 16 cases of falling. There were 29 male children and 23 female children, aged between 6 and 15 years, with an average age of (10.31 ± 1.25) years in the observation group. The accidental injuries experienced in this group were: 13 cases of fracture, 22 cases of car accidents, and 17 cases of falling injury from height. The general data of the two groups were compared (*P* > 0.05).

### Inclusion and exclusion criteria

(1) Inclusion criteria: Children who have suffered accidental injuries including fracture, car accidents, and falling; children who exhibit normal cognitive function and are in a stable condition, without any mental disorder; children with normal coagulation and immune function; family members of the children had signed an informed consent form. (2) Exclusion criteria: Children with congenital heart disease, children with acute infection, children with impairment of important organ function, and children with incomplete clinical data.

### Study method

Standard nursing care in the control group: After the children were admitted to the hospital, they were provided with a comfortable and peaceful ward environment. Furthermore, an evaluation of their personality characteristics was conducted, and psychological counseling was administered. The children were assisted in completing various routine examinations, and guidance was offered to the child or their family to encourage questions, promptly addressing any concerns. Additionally, tailored guidance on diet and medication was provided based on the preferences and condition of the child. Nursing intervention based on the stress system theory in combination with painting therapy was implemented in the observation group. The details are as follows:

#### 1) Nursing intervention based on stress system theory

(1) Stressor intensity perception intervention:

i. Continuous health education: Nurses exhibited a cordial and affable demeanor in their interactions with pediatric patients, fostering positive relationships and acquainting both the children and their families with the ward environment. They introduced attending medical staff to the children and their families, facilitating a swift acclimatization to the surroundings, thereby mitigating unfamiliarity and encouraging active cooperation. Concurrently, the nursing staff conducted comprehensive educational sessions for both children and their families, encompassing information on the medical condition, treatment plan, key cooperation points, drug efficacy, and necessary precautions. Furthermore, nurses conducted pertinent assessments of the children’s personality traits and, in accordance with individual preferences, provided toys or played cartoons to divert attention effectively. This approach aimed to create a supportive and comforting healthcare environment for pediatric patients.
ii. Relaxation therapy: (a) Abdominal Breathing: Children were directed to expand their abdomen (3 to 5 s) while inhaling, hold their breath for 1 s, compress the abdomen to achieve a concave shape (3 to 5 s) during exhalation, and hold their breath for an additional 1 s. This exercise was administered once daily, with each session lasting 10 to 15 min. (b) Muscle Relaxation: Children were instructed to sequentially relax muscles from the face down to the feet. Each muscle group was to be held in a relaxed state for 10 s, followed by a complete state of relaxation for 20 to 30 s. This exercise was to be performed once daily, with each session lasting 20 min. (c) Meditation: A serene and comfortable environment was arranged for the children, featuring soothing and aesthetically pleasing melodies like lullabies. The children were instructed to follow the music, engage in imaginative practices, practice deliberate and continuous breathing, and cultivate an awareness of the air flowing through their bodies. This meditative exercise was administered once daily, with each session lasting for 20 min.

(2) Coping style intervention:

i. One-to-One Psychological Counseling: This entailed engaging in individualized psychological counseling sessions with each child and their family. The approach involved attentive listening to the child’s feelings, analysis of reasons for negative coping, motivation to comprehend the causes of negative psychology, and facilitation of a transition to a more balanced mindset. The children were actively encouraged to express their inner thoughts and received positive affirmation.
ii. Collective Intervention: Social gatherings were arranged to foster collective activities, including having children reenact scenes from TV shows and promoting communication among the children regarding their academic and personal experiences. Additionally, leveraging children’s innate ability to imitate, those with commendable performances in the above activities were comforted and encouraged.

(3) Cognitive psychological intervention:

i. Cognitive Behavioral Therapy: The children were prompted to articulate their inner feelings, facilitating the expression and release of emotions. They were informed that experiencing stress responses is a common symptom and guided to recognize its normalcy. The therapeutic process involved encouraging the children to take the initiative in accepting these feelings. Appropriately, conversations about the accidental injury were initiated, ensuring the child’s comprehensive awareness, ultimately fostering complete acceptance. From the child’s perspective, an analysis of the causes of negative cognition was undertaken, corrections were applied, and assistance provided to help the child confront reality and approach the condition with a positive mindset.
ii. Rational Emotion Therapy: This approach involved assessing the child’s prevailing psychological challenges, with a specific focus on addressing anxiety, depressive feelings, and other emotional concerns. Collaboratively with the child, an analysis was conducted to differentiate between rational and irrational thoughts. The child was instructed to scrutinize irrational beliefs and, over time, confront them with more rational and balanced emotions.

2) Painting therapy: It is divided into four stages.

1. Warm-up Training: Prior to initiating the painting session, an introduction to the painting tools and techniques, including graffiti, shape, and color, was provided to the children. This served the purpose of stabilizing the children’s emotional state. Simultaneously, nurses employed compassionate language to alleviate any feelings of unfamiliarity, establish a trusting relationship, and prevent potential sentiments of rejection.
2. Portrait Painting: The children were guided to create portraits, focusing on rendering images of oneself, friends, and parents. Emphasis was placed on capturing physical features and expressing the affection derived from loved ones.
3. Scenic Painting: The children received instruction to depict the surrounding environment, such as supermarkets and parks. This aimed to evaluate their ability to imagine scenes, guiding them to perceive the beauty of their surroundings positively.
4. Free-Style Drawing: The children were directed to engage in free-style drawing, depicting natural scenery such as animals, flowers, plants, and mountains. This exercise aimed to stimulate their appreciation for the environment and facilitate a shift in social cognition.

### Observation indicators

1. PTSD: Before and after nursing care, the PTSD Self-evaluation Scale (PTSD-SS) [9] was used for evaluation, including subjective assessment (rating on a scale from 1 to 5 points), repeated reproduction experience (rating on a scale from 7 to 35 points), avoidance symptoms (rating on a scale from 7 to 35 points), increased alertness (rating on a scale from 6 to 30 points), and impaired social function (rating on a scale from 2 to 10 points). The lower the scores, the better. The PTSD-SS showed good reliability and validity, with an internal consistency coefficient of 0.9207, split-half reliability coefficient of 0.9539, and test-retest reliability coefficient of 0.8677.
2. Post-traumatic growth: Before and after nursing care, the children’s version of Post-Traumatic Growth Inventory (PTGI) [10] was used for assessment, including interpersonal relationships (rating on a scale from 0 to 35 points), new possibility (rating on a scale of 0–25 points), personal strength (rating on a scale from 0 to 20 points), appreciation of life (rating on a scale from 0 to 15 points), and change in spirit (rating on a scale from 0 to 10 points). The higher the score, the better. In this study, the Cronbach’s α coefficient for PTGI was 0.831, indicating good internal consistency reliability. External consistency was assessed using test-retest reliability, which was found to be 0.705.
3. Coping style: Before and after nursing care, the Medical Coping Modes Questionnaire (MCMQ) [11] was used for assessment, including Confrontation (rating on a scale from 8 to 32 points), Avoidance (rating on a scale from 7 to 28 points), and Resignation (rating on a scale from 5 to 20 points). The higher the scores of each dimension, the patients are more inclined to this coping style. The Cronbach’s α coefficients for the Chinese version of MCMQ were 0.69, 0.60, and 0.76 for the Approach, Avoidance, and Surrender dimensions, respectively, and the test-retest reliability coefficients were 0.64, 0.85, and 0.67, respectively. In this study, the Cronbach’s α coefficients for the Approach, Surrender, and Avoidance dimensions were 0.80, 0.69, and 0.79, respectively.
4. Quality of life: Before and after nursing care, the Pediatric Quality of Life Inventory Measurement Models (PedsQL4.0) [12] was used for assessment, including [physiological (rating on a scale from 0 to 32 points), emotional (rating on a scale from 0 to 20 points), social (rating on a scale from 0 to 20 points), and role (rating on a scale from 0 to 20 points) functions. The higher the score, the better. The Chinese version of the PedsQL 4.0 demonstrates good reliability and validity, with Cronbach’s α coefficients ranging from 0.74 to 0.82 for each dimension.
5. Family satisfaction: A self-made satisfaction questionnaire was used for evaluation, including service, skills, etc., and the results were categorized as ≤ 60 points: dissatisfied; 61–89 points: partially satisfied; ≥ 90 points: very satisfied. The details of the self-made satisfaction questionnaire have been provided in Supplementary Materials.

### Statistical methods

SPSS.20.0 software was used to statistically analyze the data. Based on the test results, *P* = 0.200 > 0.05, therefore, the null hypothesis was accepted, indicating that the sample was consistent with the specified distribution method, that is, consistent with a normal distribution. The count data are expressed as percentage (%), and the chi-squared test was used for comparison. The measurement data are expressed as mean ± standard deviation ($\bar x$±s), and the *t*-test was used for comparison. *P* < 0.05 indicated statistical difference.

## Results

### PTSD

The scores of each dimension in the PTSD-SS prior to nursing care were compared between the two groups (*P* > 0.05). The scores of PTSD-SS in the observation group were lower than those in the control group after nursing (*P* < 0.05) (Table 1).

**Table 1:** Comparison of PTSD between the two groups ($\bar x$±s ,points)

Groups	Subjective assessment		Repeated reproduction experience		Avoidance symptoms		Increased alertness		Impaired social function	
	Before nursing care	After nursing care	Before nursing care	After nursing care	Before nursing care	After nursing care	Before nursing care	After nursing care	Before nursing care	After nursing care
Control group (*n* = 48)	3.08 ± 0.32	2.03 ± 0.26	26.17 ± 3.52	19.49 ± 2.58	25.41 ± 2.37	16.09 ± 2.23	22.52 ± 2.27	17.18 ± 1.83	8.08 ± 0.24	6.11 ± 1.01
Observation group (*n* = 52)	3.11 ± 0.34	1.62 ± 0.20	26.21 ± 3.59	15.08 ± 1.83	25.74 ± 2.40	11.32 ± 2.11	22.59 ± 2.33	12.19 ± 1.51	8.11 ± 0.27	4.18 ± 0.83
t	0.453	8.878	0.056	9.918	0.691	10.990	0.152	14.918	0.585	10.472
P	0.651	< 0.001	0.955	< 0.001	0.491	< 0.001	0.880	< 0.001	0.560	< 0.001

* *t*-test was used for group comparison

### Post-traumatic growth

The post-traumatic growth prior to nursing care was compared between the two groups (*P* > 0.05). The scores of each dimension in the PTGI in the observation group were higher than those in the control group following nursing care (*P* < 0.05) (Table 2).

**Table 2:** Comparison of post-traumatic growth between the two groups ($\bar x$±s, points)

Groups	Interpersonal relationships		New possibility		Personal strength		Appreciation of life		Change in spirit	
	Before nursing care	After nursing care	Before nursing care	After nursing care	Before nursing care	After nursing care	Before nursing care	After nursing care	Before nursing care	After nursing care
Control group (*n* = 48)	18.54 ± 2.61	25.41 ± 3.06	12.19 ± 1.37	16.88 ± 2.11	9.61 ± 1.02	13.54 ± 1.28	6.11 ± 0.84	9.29 ± 1.03	4.20 ± 0.55	6.14 ± 0.79
Observation group (*n* = 52)	18.49 ± 2.65	29.54 ± 3.17	12.22 ± 1.40	20.54 ± 2.73	9.63 ± 1.07	16.10 ± 1.37	6.15 ± 0.87	11.05 ± 1.12	4.28 ± 0.49	8.20 ± 0.98
t	0.095	6.618	0.108	7.456	0.096	9.634	0.234	8.158	0.769	11.513
P	0.925	< 0.001	0.914	< 0.001	0.924	< 0.001	0.816	< 0.001	0.444	< 0.001

* *t*-test was used for group comparison

### Coping style

The coping style prior to nursing care were compared between the two groups (*P* > 0.05). The Confrontation scores of MCMQ in the observation group were higher than those in the control group prior to nursing care. The Avoidance and Resignation scores were lower than those in the control group (*P* < 0.05) (Table 3).

**Table 3:** Comparison of coping styles between the two groups ($\bar x$±s, points)

Groups	Confrontation		Avoidance		Resignation	
	Before nursing care	After nursing care	Before nursing care	After nursing care	Before nursing care	After nursing care
Control group (*n* = 48)	15.71 ± 2.51	18.21 ± 3.10	16.18 ± 2.16	13.51 ± 1.40	13.40 ± 1.61	11.60 ± 2.12
Observation group (*n* = 52)	15.24 ± 2.22	21.53 ± 3.41	16.81 ± 2.34	11.18 ± 1.53	13.62 ± 1.84	9.11 ± 1.43
t	0.994	5.080	1.396	7.924	0.634	6.933
P	0.323	< 0.001	0.166	< 0.001	0.528	< 0.001

* *t*-test was used for group comparison

### Quality of life

The quality of life prior to nursing care was compared between the two groups (*P* > 0.05). The scores of each dimension in PedsQL4.0 in the observation group were higher than those in the control group following nursing care (*P* < 0.05) (Table 4).

**Table 4:** Comparison of quality of life between the two groups ($\bar x$±s, points)

Groups	Physiological function		Emotional function			Social function			Role function		
	Before nursing care	After nursing care	Before nursing care		After nursing care	Before nursing care		After nursing care	Before nursing care		After nursing care
Control group (*n* = 48)	12.09 ± 2.05	18.91 ± 3.52	8.89 ± 1.22		12.14 ± 2.03	7.10 ± 1.04		11.52 ± 1.25	8.50 ± 1.12		13.49 ± 2.67
Observation group (*n* = 52)	12.08 ± 2.01	26.30 ± 3.67	8.91 ± 1.23		16.35 ± 2.36	7.04 ± 1.01		15.91 ± 2.50	8.47 ± 1.08		17.12 ± 2.30
t	0.025	10.259	0.082		9.526	0.293		10.964	0.136		7.300
P	0.980	< 0.001	0.935		< 0.001	0.771		< 0.001	0.892		< 0.001

* *t*-test was used for group comparison

### Family satisfaction

The observation group exhibited family satisfaction that was higher than that of the control group (*P* < 0.05) (Table 5).

**Table 5:** Comparison of family satisfaction between the two groups (%)

Groups	Very satisfied	Partially satisfied	Dissatisfied	Total satisfaction
Control group (*n* = 48)	15(31.25)	24(50.00)	9(18.75)	39(81.25)
Observation group (*n* = 52)	21(40.38)	29(55.77)	2(3.85)	50(96.15)
χ^2^				5.663
P				0.017

* *t*-test was used for group comparison

## Discussion

PTSD is prevalent in children following accidental injuries. If effective interventions are not taken, it can jeopardize their mental health, hinder future independence, and greatly affect their quality of life [13–15]. Therefore, scientific and effective measures should be taken to help children with accidental injuries to reduce traumatic stress and promote post-traumatic growth.

Standard nursing care focuses on accidental injuries, with nursing staff primarily providing basic care without specifically evaluating the risk of PTSD in children. This lack of assessment hinders the implementation of effective nursing interventions, rendering them inadequate for meeting the developmental needs of children and their families. Consequently, the clinical application of these interventions is limited [16]. In this study, following nursing intervention, the observation group exhibited lower scores in each dimension of PTSD-SS compared to the control group. Additionally, the observation group had higher scores in each dimension of PTGI and MCMQ Confrontation scores, as well as lower scores in Avoidance and Resignation compared to the control group. Furthermore, the observation group had higher scores in each dimension of PedsQL4.0 and the family satisfaction was higher than that in the control group. These findings suggest that the nursing intervention based on stress system theory combined with painting therapy have significant effects on children with PTSD after accidental injury. This is because the stress system theory posits that the intensity of stressors is a crucial mediating factor influencing stress responses. Exposure to a visually pleasing environment in children can heighten brain excitability, evoke emotions, mitigate adverse effects stemming from social and psychological factors, and enhance stress tolerance. Consequently, this intervention effectively contributes to the amelioration of PTSD [17, 18]. Meanwhile, muscle relaxation, abdominal breathing, and meditation can help children reduce the physical and mental response to stressful events, alleviate negative emotions, and reduce pain response during treatment, thereby speeding up physical and mental recovery, and improving quality of life. The coping style of an individual reflects their behavioral and cognitive responses to stressful events. A positive coping style has a buffering effect on the impact of stress and promotes disease stability. Günay et al. found that the activity of making jewelry from beads was effective in reducing the state and trait anxiety levels of children with cancer [4]. Similarly, Sarman et al. reported that Goldfish intervention was found to be effective in decreasing the state anxiety and fear levels and increasing the psychological and emotional well-being levels of the children in the study group [7]. However, a negative coping style significantly aggravates the stress in the patient and has an adverse impact on the development and prognosis of the disease. For instance, Hao et al. showed that negative coping styles intensify the negative effect of illness uncertainty on demoralization in patients with breast cancer [19]. In this study, we addressed the coping style and cognitive behavior according to the stress system theory. This intervention helped children to correct their misconceptions and actively accept and confront their current emotions. By changing their negative coping styles, it is possible to effectively reduce psychological trauma and stress response and promote post-traumatic growth. Painting therapy, an essential aspect of art therapy, mainly uses non-verbal symbols to promote effective interaction and expression of inner thoughts through painting. Its primary purpose is to achieve catharsis of negative emotions [20]. In this study, painting therapy was divided into four stages: warm-up training, portrait painting, scenic painting, and free-style painting. A quiet and comfortable painting environment was provided for the children, so that they could concentrate on painting consciously, which was conducive to diverting their attention from the condition and reducing the interference of negative emotions. Meanwhile, the traumatic stress response of children can be expressed in the form of paintings, which can achieve the purpose of reducing PTSD and promoting post-traumatic growth.

### Strengths and limitations

Previous studies primarily used methods such as health education, need-oriented personalized care, and game therapy for the physical and mental intervention of children, which require a high level of language expression, cognitive ability, and social communication skills. Due to the lack of self-control in some children and their strong dependence on parents, implementation can be challenging. In this study, we examined the impact of a nursing intervention based on stress system theory, coupled with painting therapy. During the painting process, children can express repressed feelings and conflicts from their subconscious, fully revealing their true inner emotions and reducing negative emotions. Compared to other intervention methods, painting therapy is not limited by the child’s language, age, or educational level. It is easy to source materials, practical, and enjoyable. Additionally, painting therapy does not emphasize the aesthetic quality of the artwork or drawing skills, allowing for a free creation process and high participation from the children.

The limitations of this study should also be acknowledged. Firstly, this study was a single-center study, with a small sample size. Future studies with a multicenter and randomized controlled trial design, as well as longer follow-up, are needed to validate our findings and to explore the long-term effects of this approach in different settings. Secondly, the different cultural backgrounds of the subjects’ families may introduce bias into the results.

## Conclusion

To sum up, our study showed that implementation of nursing intervention based on stress system theory in combination with painting therapy effectively alleviated PTSD in children who have experienced accidental injury. This approach not only assists them in copying with their condition but also aid them to adopt a positive attitude. Consequently, it fosters post-traumatic growth, improves their quality of life, and boosts family satisfaction levels. These outcomes underscore the substantial clinical value of this approach and suggest its potential for broader application in the population with similar conditions.

## Data Availability

The datasets generated and analysed during the current study are not publicly available but are available from the corresponding author （Xing Yuan）on reasonable request.

## Abbreviations

PTSD: Posttraumatic Stress Disorder
PTSD-SS: Posttraumatic Stress Disorder Selfrating Scale
PTGI: Posttraumatic Growth Inventory
MCMQ: Medical CopingModes Questionnaire
PedsQL: Pediatric Quality of Life Inventory
